# A Comparative Study of Deaths Due to the COVID-19 Pandemic During the First and Second Waves in a Tertiary Care Center of a Rural Area in South India

**DOI:** 10.7759/cureus.52184

**Published:** 2024-01-12

**Authors:** Rahul Navab, Anil R, Uma M A, Dhananjaya P E, Sangeetha Kamatchi, Visweswara Reddy Yeragudi jangamareddy

**Affiliations:** 1 Internal Medicine, People's Education Society Institute of Medical Sciences and Research, Kuppam, IND; 2 Preventive Medicine, People's Education Society Institute of Medical Sciences and Research, Kuppam, IND; 3 Quality Management Services, People's Education Society Institute of Medical Sciences and Research, Kuppam, IND; 4 Internal Medicine, People’s Education Society Institute of Medical Sciences and Research, Kuppam, IND

**Keywords:** age group, india, comorbid, deaths, covid-19

## Abstract

Background

The coronavirus disease 2019 (COVID-19) pandemic affected life and livelihood worldwide, including India, with over five million deaths recorded over two years. In the present study, our objective was to analyze the COVID-19 deaths during the first and second waves in relation to demographic factors and comorbid conditions.

Methods

This was a hospital-based, retrospective comparative study of COVID-19 deaths that occurred in our hospital during the first and second waves of the COVID-19 pandemic. A total of 210 (6.69%) deaths recorded during both waves of the pandemic were analyzed. Microsoft Excel sheets (Microsoft Corporation, Redmond, WA, USA) were used to collect data from the medical records section, and the data were compiled. Descriptive statistics were used and analyzed using SPSS version 21 (IBM Corp., Armonk, NY, USA).

Results

Out of 3136 inpatients, mortality was 6.69% (n=210). Out of 210 deaths recorded in the study, 34 (2.25%) and 176 (10.7%) were during the first and second waves of the pandemic, respectively. The most common age group affected during the two waves was 50-75 years (67.6% & 47.7%; n=23 & n=84). People from urban (52.9%; n=18) and rural (67%; n=118) backgrounds were affected more during the first and second waves, respectively. Males were affected more (72.8%; n=153) in both waves. Age group (P=0.009) and locality (P=0.026) were statistically significant factors associated with mortality in the two COVID-19 waves. The time interval from admission in the hospital to death was less than seven days in both waves (70.5% & 69.8%; n=24 & n=123). A large number of subjects died after 48 hours of admission during both waves (70% (n=24 & n=124) in each wave). More than half of the subjects who died (52.9% (n=18) & 59% (n=104)) had comorbid conditions in both waves. Diabetes mellitus (17.6%; n=6) and hypertension (23.5%; n=8) were the most common comorbid conditions during the first wave of the pandemic while diabetes mellitus (30.6%; n=54) alone was the most common during the second wave.

Conclusion

The findings of this study stress the importance of considering demographic factors and geographic locations in understanding the impact of COVID-19, providing valuable inputs for public health interventions and resource allocation in response to similar pandemics.

## Introduction

A global pandemic caused by the coronavirus wasn’t known to the world until December 2019, when a cluster of pneumonia-like symptoms was reported from Wuhan, China [1]. India reported its first confirmed case of severe acute respiratory syndrome coronavirus-2 (SARS-CoV-2) on January 30, 2020 [2]. By January 2022, there were 4.13 crore cases and 4,95,050 deaths reported in India [3]. There were about 14,460 causalities reported in the state of Andhra Pradesh, India, by December 2021 [4]. A study regarding the coronavirus disease 2019 (COVID-19) pandemic during March-October 2020 in India, which included nine states (Delhi, Gujarat, West Bengal, Uttar Pradesh, Andhra Pradesh, Maharashtra, Karnataka, Tamil Nadu, and Telangana) recorded 80% of all infected cases and 61% of all deaths [5]. There was little variation in the rate of fatalities between the first and second waves of the pandemic, according to the Indian Council of Medical Research Director General [6]. Factors such as economic condition, diabetes, hypertension, overweight, obesity, and literacy were significantly associated with COVID-19 mortality [7].

Therefore, this study was undertaken to analyze deaths due to the COVID-19 pandemic during the first and second waves; it focused on demographic factors and comorbid conditions, which may guide and prepare us for the future.

## Materials and methods

This was a single-center retrospective study and data were collected from February 2020 to January 2022.

The study was carried out at a government-recognized center for the management of COVID-19 patients in the southern area of Chittoor district; treatment was provided free of cost to all health cardholders between February 2020 and January 2022. A total of 3136 cases of COVID-19 were treated (first wave = 1506 and second wave = 1630), 2926 (93.3%) patients recovered, and 210 deaths (6.69%) were recorded.

All 210 (6.69%) deaths due to COVID-19 across all age groups were included in the study, with 34 (2.25%) occurring during the first wave of the pandemic (February to December 2020) and 176 (10.7%) during the second wave (March to January 2022). Data were obtained from the medical records department section of our institution after getting the necessary permissions from the college and hospital authorities. A Microsoft Excel sheet (Microsoft Corporation, Redmond, WA, USA) was prepared to collect data, including factors like gender, age, region, co-morbidities like diabetes mellitus, hypertension, chronic kidney disease, coronary artery disease, duration of hospital stay, and outcome.

The data were compiled and analyzed using SPSS version 21. Descriptive statistics were used for categorical data in the form of percentages. Inferential statistics, such as the chi-square test, were used for analyzing statistical associations. A P-value of <0.05 was considered statistically significant.

Ethical clearance was provided by the institutional ethical committee of PES Institute of Medical Sciences and Research (approval number: PESIMSR/IHEC/129-21).

## Results

**Figure 1:**
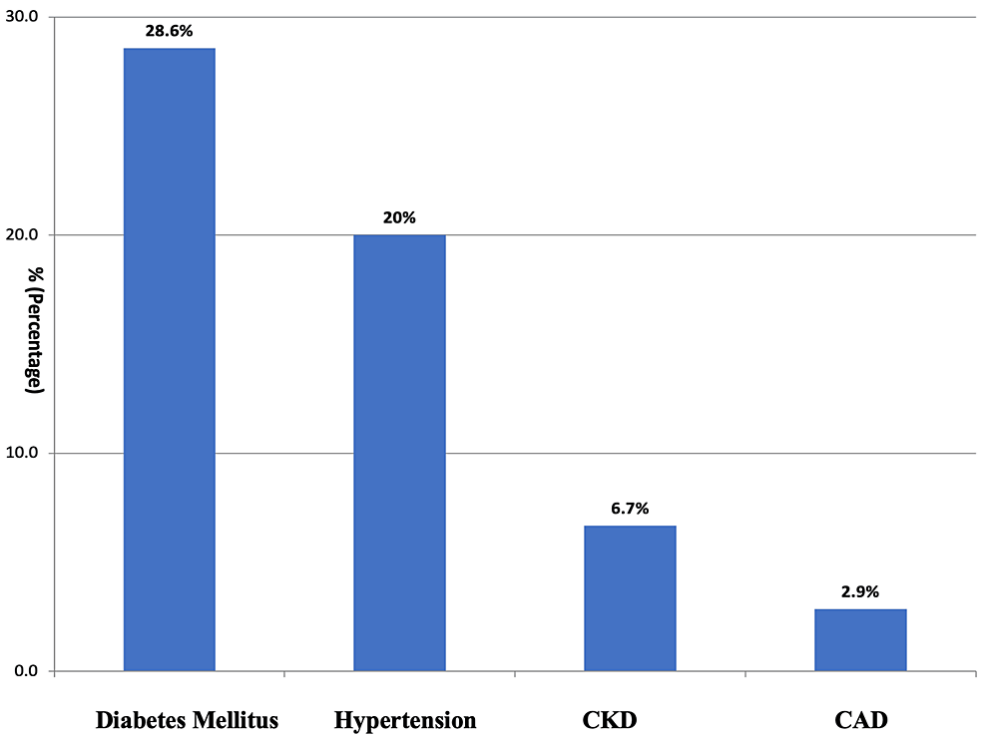
The different co-morbid conditions present among the deceased % = percentage, CKD = chronic kidney disease, CAD = coronary artery disease

The total number of patients admitted and treated was 3136, among which 210 (6.69%) deaths were recorded. Out of 210 deaths in the study, 34 (2.25%) were during the first wave of the COVID-19 pandemic, from February to December 2020, and 176 (10.7%) died during the second wave of the pandemic, from March to January 2022. Among the 210 deaths, 72.8% (n=153) were males and 27.1% (n=57) were females. The majority (50.9%; n=107) of the deaths were seen in the age group of 51-75 years. The median age at death in both waves was 54 years. Deaths were seen more among the subjects from rural areas (63.8%; n=134) than compared to urban areas (36.1%; n=76). Among the total deaths, patients had more comorbid conditions during the second wave (59%; n=104) compared to the first wave(52.9%; n=18). The duration of hospital stay in 70% (n=147) of deaths was less than seven days. Among the 210 (6.69%) deaths in the hospital, 29.5% (n=62) died within 48 hours (Table 1). The different co-morbid conditions present among the deceased are shown in Figure 1.

**Table 1:** Demographic details and comorbidities in COVID-19 deaths in both waves combined N: number, %: percentage

Gender		N	%
Female		57	27.1
Male		153	72.8
Age group (in years)			
<25		8	3.8
26-50		75	35.7
51-75		107	50.9
>76		20	9.5
Region			
Rural		134	63.8
Urban		76	36.1
Comorbidities			
Present	Hypertension	42	34.4
	Diabetes mellitus	62	50.8
	Chronic kidney disease	14	11.5
	Coronary artery disease	4	3.3
Absent		88	41.9
Duration of hospital stay			
<7 days		147	70
7-14 days		53	25.2
>14 days		10	4.7
Death occurrence			
<24 hours		25	11.9
24-48 hours		37	17.6
>48 hours		148	70.4
Total		210	100

In both waves, deaths were more among the males (64.7% and 74.4%; n=22 & n=131) and there was no statistically significant association between gender and the COVID-19 waves. In the first wave, the majority (85.2%; n=29) of the deaths were noticed in the age group of more than 50 years, whereas in the second wave, only 55.6% (n=98) deaths were in the age group of more than 50 years. The median age at death in the first and second waves was 65 years and 52 years, respectively. A statistically significant association was noted between the age group and the waves of COVID-19 (P=0.009). Almost equal proportions of deaths were seen among the rural (47%) (n=16) & urban area (52.9%) (n=18) subjects during the first wave. During the second wave, deaths were higher (67%) (n=118) in the people who belonged to rural areas, and that was statistically significant (P=0.026). In both waves, the majority (70.5% & 69.8%; n=24 & n=123) of the deaths occurred within seven days.

More than 50% of the deaths that occurred during both waves have an associated comorbid condition, which was 52.9% (n=18) in the first wave and 59% (n=104) in the second wave. Co-morbid conditions like diabetes (28.6%; n=60) and hypertension (20%; n=42) were higher among the people who died during the COVID first and second waves, as shown in (Table 2).

**Table 2:** A comparison of deaths during the first and second waves of COVID-19 n: number, %: percentage, CKD: chronic kidney disease, CAD: coronary artery disease, * P-value of <0.05 is considered statistically significant

Associated Factors	1^st^ Wave Number (Percentage)	2^nd^ Wave Number (Percentage)	Total Number (Percentage)	Chi-Square Score (P-value)
Gender				
Male	22 (64.7%)	131 (74.4%)	153 (72.8%)	1.3630 (0.243)
Female	12 (35.2%)	45 (25.5%)	57 (27.1%)	
Age group in years				
<25	1 (2.9%)	7 (3.9%)	8 (3.8%)	11.6256 (0.009)*
26-50	4 (11.7%)	71 (40.3%)	75 (35.7%)	
51-75	23 (67.6%)	84 (47.7%)	107 (50.9%)	
>76	6 (17.6%)	14 (7.9%)	20 (9.5%)	
Locality				
Rural	16 (47%)	118 (67%)	134 (63.8%)	4.9291 (0.026)*
Urban	18 (52.9%)	58 (32.9%)	76 (36.1%)	
Duration of stay				
<7 days	24 (70.5%)	123 (69.8%)	147 (70%)	0.1559 (0.925)
7-14 days	8 (23.5%)	45 (25.5%)	53 (25.2%)	
>14 days	2 (5.8%)	8 (4.5%)	10 (4.7%)	
Time lapse from admission to death				
<24 hours	3 (8.8%)	22 (12.5%)	25 (11.9%)	0.5266 (0.768)
24-48 hours	7 (20.5%)	30 (17%)	37 (17.6%)	
>48 hours	24 (70%)	124 (70%)	148 (70.4%)	
Comorbidities				
Present	18 (52.9%)	104 (59%)	122 (58.1%)	0.4427 (0.506)
Absent	16 (47%)	72 (40.9%)	88 (41.9%)	
Diabetes mellitus				
Absent	28 (82.3%)	122 (69.3%)	150 (71.4%)	2.372 (0.305)
Present	6 (17.6%)	54 (30.6%)	60 (28.6%)	
Hypertension				
Absent	26 (76.4%)	142 (80.6%)	168 (80%)	0.3158 (0.574)
Present	8 (23.5%)	34 (19.3%)	42 (20%)	
CKD				
Absent	32 (94.1%)	164 (93.1%)	196 (93.3%)	0.0401 (0.841)
Present	2 (5.8%)	12 (6.8%)	14 (6.7%)	
CAD				
Absent	32 (94%)	172 (97.7%)	204 (97.1%)	3.118 (0.210)
Present	2 (5.8%)	4 (2.2%)	6 (2.9%)	
Total	34	176	210	

## Discussion

​​​​​​The number of death cases recorded in our hospital was 34 (2.25%) during the first wave, i.e., February 2020 to December 2020, and 176 (10.7%) during the second wave, i.e., March 2021 to January 2022. During the first wave, many hospitals and healthcare professionals were on a learning curve, as it was a new disease and experience. Although, during the second wave, more hospital admissions were possible, more deaths occurred, possibly due to the different strain, higher burden of admission, and unavailability of oxygen.

The present study was carried out to compare the COVID-19 deaths during the first and second waves in relation to demographic factors and comorbid conditions among various age groups. In both waves, deaths among the males were greater when compared to females (Males: Females = 72.8% (n=153): 27.1% (n=57)). As per the national registry, males were more (65.4% & 63.7%) affected in both waves as seen in this study [8]. In the current study, subjects belonging to the age group of 50-75 years were affected majorly in both waves (67.6% (n=23) & 47.7%(n=84) ). The median age of death due to COVID-19 was 64 years and 52 years during the first and second waves, respectively. The median age group affected was 47-59 years in one of the studies conducted during the first wave of the COVID-19 pandemic [9]. The Indian Council of Medical Research Director General noted that 70% of infected patients were more than 40 years old in both waves [6]. In one of the studies conducted in our neighboring state - Tamil Nadu, the median age of death was found to be 64 years, which is similar to our study [10]. Among the subjects who died during the first wave, 17.6% (n=6) were aged more than 75 years, which is identical to a study conducted in two Indian states (Tamil Nadu and Andhra Pradesh) [11]. There was a statistically significant association seen between the age group and the waves of COVID-19 (P=0.009). In the first wave of the pandemic, subjects from urban backgrounds were affected more (52.9%; n=18) because of high population density [12], whereas, in the second wave, more subjects (67%; n=118) from the rural area succumbed to the deadly virus. Because of the older population, higher comorbidities, and lack of access to healthcare, rural areas were particularly vulnerable [12].

The time interval from admission to the hospital to death was less than seven days in both waves (70.5% (n=24) vs 69.8% (n=123)). A study conducted in the states of Tamil Nadu and Andhra Pradesh revealed that half of the cases of deaths occurred within six days of the testing [11]. In this study, 29.5% (n=62) of the total deaths happened within 48 hours after admission, which is lower by comparison to a study that reported 49.8% of the deaths happened between 48 and 72 hours [10]. This could be attributed to better care given in our institution, as per central and state government guidelines and protocols.

Deaths (52.9% (n=18 and 59% (n=104)) reported during the first and second waves of the pandemic, respectively, had comorbidities. During the first wave of the pandemic, diabetes mellitus (17.6%; n=6) and hypertension (23.5%; n=8) were the most prevalent comorbid conditions, followed by chronic kidney disease (5.8%; n=2) and coronary artery disease (5.8%; n=2). A study conducted by Cunningham JW et al., during the first wave of the pandemic, revealed that there was a higher risk of adverse events associated with comorbid conditions like obesity, hypertension, and diabetes [13]. A study conducted in Tamil Nadu showed that diabetes (66%), followed by hypertension (54%), coronary artery disease (18%), and chronic kidney disease (15%) were the common comorbid conditions reported [10]. A study, on causes of COVID-19 deaths and comorbidities in hospitalized patients, revealed arterial hypertension (65.4%) was the most prevalent comorbid condition during the first wave of the pandemic [14]. In the second wave of the pandemic, diabetes mellitus (30.6%; n=54) was the most prevalent comorbidity, followed by hypertension (19.3%; n=34), chronic kidney disease (6.8%; n=12), and coronary artery disease (2.2%) (n=4) among the 176 deaths. There is not much literature available to compare the comorbidities in the death cases during the second wave of the COVID-19 pandemic. However, among the death cases, multiple comorbidities were noted. Both diabetes mellitus and hypertension were found in 14.7% (n=5) and 13.6% (n=24) during the first and second waves, respectively, and both hypertension and chronic kidney disease were found in 2.9% (n=1) and 3.4% (n=6) during the first and second waves, respectively.

This study was a retrospective and hospital-based single-center study. The center where this study was conducted is situated in a rural area. Considering these limitations, a large sample size covering a huge geographical region will give more valuable insights to this study. This study hasn’t compared the comorbid conditions in patients who did not die.

## Conclusions

During the first and second waves of the COVID-19 pandemic, death rates marked a fivefold increase. Both waves highlighted the impact on different adult age groups, with significant regional variations. The prevalence of deaths was high among males, individuals aged 51-75, and urban subjects during the first wave, while the second wave saw a higher toll among males, the same age group, and rural subjects. A majority of deaths occurred more than 48 hours after admission and treatment, highlighting the critical need for timely interventions.
